# Comment on “Prediction of the 1-Year Risk of Incident Lung Cancer: Prospective Study Using Electronic Health Records from the State of Maine”

**DOI:** 10.2196/14944

**Published:** 2020-09-15

**Authors:** Jamal Rahmani, Roya Karimi, Yousef Khani, Siamak Sabour

**Affiliations:** 1 Department of Clinical Epidemiology School of Health and Safety Shahid Beheshti University of Medical Sciences Tehran Iran; 2 Safety Promotions and Injury Prevention Research Centre Shahid Beheshti University of Medical Sciences Tehran Iran

**Keywords:** prediction, area under the curve, AUC, lung cancer

We read the recent article by Wang et al [1] with great interest. This paper was published in 2019 in the *Journal of Medical Internet Research*. The authors aimed to develop and validate a prospective risk prediction model to identify patients at risk of new incident lung cancer within the next 1 year in the general population. They used data from individual patient electronic health records (EHRs), which was extracted from the Maine Health Information Exchange network. The Extreme Gradient Boosting (XGBoost) algorithm was adopted to build the model, and the authors reported an area under the curve (AUC) of 0.88 (95% CI 0.87-0.88) for their model validation, according to a prospective cohort data. Finally, the authors concluded that their model was able to identify statewide, high-risk patients.

Risk prediction models are effectively useful due to their role in decision making. However, there are some methodological commentaries that we would like to mention. First, AUC is an appropriate measure for assessing discrimination. Discrimination is defined as the ability to distinguish events versus nonevents. However, it assumes that two persons are randomly selected—one who will develop the disease and one who will not. AUC assigns a higher probability of an outcome to the one who will develop the disease. A c-index value of 0.5 expresses a random chance; however, the usual c-index for a prediction model is 0.60 to 0.85. This range can be changeable under different conditions. What we should always consider about the AUC measure is that a high value of AUC discerns excellent discrimination, but it can also reflect a situation with limited relevance. This situation might arise because the variable is related to the diagnostic or early onset of the disease instead of prediction [2,3]. Furthermore, the receiver operating characteristic (ROC) would be a good tool for binary classification, but it is not instrumental for risk stratification. For risk stratification (low- and high-risk bins), the sensitivity in low and high specificity, and positive predictive value (PPV) in high-risk bins, are more discriminating parameters for the ability of the algorithm.

Second, there are several types of external validation such as validation in more recent patients (temporal validation), in other places (geographic validation), or by other investigators at other sites (fully independent validation). Having two exemplary data sets with huge sample sizes, it would be suggestible to test the above-mentioned external validity. Moreover, internal validation is a necessary part of model development. It determines the reproducibility of a developed prediction model for the derivative sample and prevents the over-interpretation of the data. Resampling techniques, such as cross-validation and bootstrapping, can be performed; bootstrap validation, in particular, appears to be the most attractive option for obtaining stable optimism-corrected estimates [2]. Furthermore, it is of importance that the authors add the validation of data production in the real world after deployment, since it would be more revealing due to the unexpected data challenges encountered during real-time usage by clinical providers.

Third, a mistake that is very common occurs when referring to statistically significant *P* values. A *P* value depends on statistical, instead of clinical, logic; thus, researchers should consider judging outputs based on effect size, rather than *P* value.

A further common issue is missing data that can influence the model development. Missing data often follow a nonrandom pattern, where there is an explanation and cause behind it. If all missing values are removed, the cause and explanation will be lost, which may affect the conclusion and the model development. To generate the model, multivariable regression techniques usually use as a stepwise model (backward is more preferable), and concomitantly checking the Akaike information criterion can help us to decide if the model fits well enough.

Finally, it is important to investigate the interactions between variables in prediction studies. Developing a model, score, or index without considering interactions among variables may elicit changes to the prediction in the real world and lead to misleading messages [3-5].

## Abbreviations

AUC: area under the curve
EHR: electronic health record
PPV: positive predictive value
ROC: receiver operating characteristic
